# Carbon-Ion Radiotherapy for Hepatocellular Carcinoma: Current Status and Future Prospects: A Narrative Review

**DOI:** 10.3390/jcm14176107

**Published:** 2025-08-29

**Authors:** Reina Sasaki-Tanaka, Hiroyuki Abe, Tomoaki Yoshida, Yusuke Watanabe, Naruhiro Kimura, Takeshi Yokoo, Akira Sakamaki, Hiroteru Kamimura, Kenya Kamimura, Tatsuo Kanda, Shuji Terai

**Affiliations:** 1 Division of Gastroenterology and Hepatology, Graduate School of Medical and Dental Sciences, Niigata University, Niigata 951-9510, Japan; hiroyukiabe@med.niigata-u.ac.jp (H.A.); tomomot.1105@gmail.com (T.Y.); ywatanabe19840421@med.niigata-u.ac.jp (Y.W.); nkimura@med.niigata-u.ac.jp (N.K.); t-yokoo@med.niigata-u.ac.jp (T.Y.); saka-a@med.niigata-u.ac.jp (A.S.); hiroteruk@med.niigata-u.ac.jp (H.K.); terais.med@niigata-u.ac.jp (S.T.); 2 Department of General Medicine, Niigata University School of Medicine, Niigata 951-9510, Japan; kenya-k@med.niigata-u.ac.jp; 3 Division of Gastroenterology and Hepatology, Uonuma Institute of Community Medicine, Niigata University Medical and Dental Hospital, Uonuma Kikan Hospital, Minamiuonuma 949-7302, Japan

**Keywords:** hepatocellular carcinoma, radiation therapy, particle beam therapy, local control, overall survival

## Abstract

Because hepatocellular carcinoma (HCC) is a radiosensitive cancer, radiation therapy has been used for the treatment of HCC; however, external beam therapies are currently not described in most of the guidelines for the treatment of HCC. External beam therapies include photon beam therapies and particle beam therapies, which are composed of X-rays or gamma rays and beams of carbon ions or protons, respectively. The focus of this narrative review is carbon-ion radiotherapy (C-ion RT). C-ion RT is well tolerated by elderly patients with HCC and/or sarcopenic patients. In general, a single HCC greater than 30 mm is a good indication for C-ion RT in patients with Child Grade A/B or ALBI Grade 1/2. The local control rates and overall survival rates at 5 years after C-ion RT for HCCs larger than 30 mm are excellent, with fewer adverse events, such as radiation-induced liver damage. Advanced HCC with portal vein tumor thrombus is also an indication for C-ion RT in certain selected patients. C-ion RT is a promising therapeutic option for patients with HCC.

## 1. Introduction

Hepatocellular carcinoma (HCC) is one of the most common malignancies and the third leading cause of cancer death worldwide in 2020 [1]. The major risk factors for HCC are chronic hepatitis B virus (HBV) or hepatitis C virus (HCV) infection, alcohol-related liver disease (ALD), or metabolic dysfunction associated with steatotic liver disease (MASLD) [2]. In recent years, the number of new cases of HCC has increased in Europe and North America because of the increase in MASLD [3,4]. There are several risk factors for reduced HCC survival, including primary liver tumor size, lymph node involvement, metastasis, HCV and HBV coinfections, race, age, and type of treatment [5,6,7].

Depending on the tumor stage, degree of liver function, and patient performance status, treatment options, including surgical, locoregional, and systemic therapies, should be selected for patients with HCC. HCC often appears in patients with advanced liver fibrosis or cirrhosis. It is important to adequately preserve liver function. In general, liver function, tumor burden/number, and vascular invasion and performance of each patient with HCC affect the selection of therapies for HCC.

Radiofrequency ablation (RFA), surgical resection, and liver transplantation are the treatments of choice in the early stages, whereas transcatheter arterial chemoembolization (TACE) is used in the intermediate stage, and systemic treatments, which were developed during recent decades, are used for advanced stages, according to the guidelines from the American Association for the Study of Liver Diseases (AASLD), the European Association for the Study of the Liver (EASL), the Asian Pacific Association for the Study of the Liver (APASL), and the Japan Society of Hepatology [8,9,10,11].

High tumor recurrence rates continue to limit long-term survival [12,13]. Recent real-world meta-analysis of combination therapy of atezolizumab plus bevacizumab for unresectable HCC showed that 24-month overall survival (OS) rate and 24-month progression-free survival (PFS) were 39% (*n* = 1556; 95% CI: 31–49; I^2^ = 90%) and 25% (*n* = 732; 95% CI: 12–45; I^2^ = 95%), respectively [14]. The 36-month OS rate (95% CI) with combination therapy of tremelimumab plus durvalumab for unresectable HCC was 36.2% (28.1–46.7) [15]. In the era of immunotherapy, 1640 HCC patients with overall treatment strategy showed that the 6, 12, and 18 months OS rates were 84.9% (95% CI: 82.8–87), 76.7% (95% CI: 74.2–79.2), and 69.3% (95% CI: 66.4–72.3), by Carcinome HépatocellulaIrE en France (CHIEF) [16].

External beam radiotherapy includes photon beam therapy which uses X-rays or gamma rays, and particle beam therapy which uses beams of protons and heavy ions. Stereotactic body radiation therapy (SBRT) using image-guided radiation therapy (IGRT) strategies and motion management strategies [17] have been reported to have promising prospective results in early stage small HCC [18]. In contrast to SBRT, particle beam therapy, including carbon-ion radiotherapy (C-ion RT)/heavy ion beam therapy (HIBT) and proton beam therapy (PBT), have been used for focal treatment options as radical therapy for HCC patients with HCC tumors ≥4 cm and relatively limited unresectable or unable to receive other local therapies. They have been covered under Japan’s Health Insurance System since April 2022, although multicenter randomized controlled studies (RCTs) are still lacking. Representative external beam therapies for HCC are shown in Table 1.

In this review, we focused on C-ion RT for HCC and searched the English literature concerning particle beam therapy to clarify its safety and effectiveness, via PubMed for the period from 2015 to July 2025.

## 2. Carbon-Ion Radiotherapy (C-Ion RT)

Unlike conventional photon modalities, such as SBRT, particle beam therapy offers unique physical and biological advantages. Beams of ionized particles are accelerated and concentrate doses to targets while reducing adverse events [19]. The application of accelerated, charged particles in radiotherapy is evidenced in the work of Robert Rathnub Wilson of the Lawrence Berkeley Laboratory at the University of California, Berkeley (Berkeley, CA, USA) in 1946 [20]. Two types of particle beam therapies are currently used for liver cancer treatment: C-ion RT and PBT. Carbon ion beams constitute the primary delivery method of heavy ion radiotherapy. The initial clinical implementations were conducted at the University of Tsukuba for PBT and in the National Institute of Radiological Sciences in Japan for C-ion RT in 1983 and 1994, respectively [21,22].

**Figure 1:**
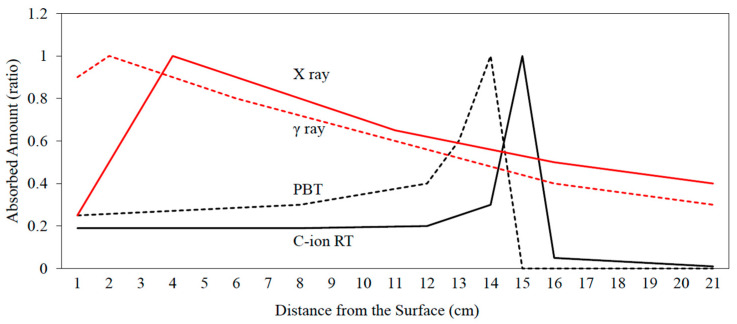
**Depth–dose distribution for electromagnetic radiation and monoenergetic Bragg curves for particle radiation.** Black solid line: carbon-ion radiotherapy (C-ion RT); black dotted line: proton beam therapy (PBT); red solid line: X-ray; red dotted line: γ ray.

Charged particle beams exhibit a characteristic depth–dose profile (Bragg curve) with a finite range in tissue and deposit most of their energy at the end of their track, termed the “Bragg peak” (Figure 1) [23,24]. The charged particle deposits only a small dose in the entrance region (plateau) of the Bragg curve and then spreads out the Bragg peak distribution, and the dose drops off quickly beyond the Bragg peak, which leads to minimal damage to critical organs behind the target [23,24]. Bragg peak distribution of linear energy transfer (LET) refers to the rate of energy loss experienced by particle beams as they penetrate tissue, which leads to effective delivery to the target [25]. For clinical applications, the range of the peak is shifted to form a so-called spread-out Bragg peak, which generates an appropriately sized treatment field with high doses [26].

Although both charged particle beams allow for highly conformal tumor irradiation, there are some differences between PBT and C-ion RT. First, the transverse scattering and range straggling of C-ion RT are relatively small compared with those of PBT with an increasing mass of particles up to approximately 160 mm depth in water, which means a smaller beam halo [27]. Although the width of the Bragg curve increases with increasing particle energy, the width of the Bragg curve decreases as the particle mass increases, which is one of the advantages of C-ion RT, compared to PBT [27].

Second, the relative biological effectiveness (RBE), which is determined by a biological endpoint such as the endpoint of cell survival for in vitro experiments, is fixed to a value of 1.1 for all PBTs and 1 for photon; in contrast, the RBE of carbon ions is not a constant value [28]. Proton beams and photons are low-LET radiations; in contrast, carbon ion beams are high-LET radiations that exhibit dense ionization compared with low-LET. Compared with low-LET radiation, high-LET radiation tends to have a high RBE. The RBE of carbon ions depends on their position within the treatment beam and tends to increase as they penetrate further into the target lesion [29]. The RBE of carbon ions varies much more for different physical factors, such as LETs and dose levels, biological factors, such as tissue type, different endpoints, and parameters [30]. The RBE must be calculated by biomathematical models and considered in the dose prescription.

Third, C-ion RT and PBT are thought to induce clustered DNA damage [31,32]; however, there is a difference in repair ability. Clustered DNA damage, which is the major factor responsible for ionizing radiation-induced cell death, is defined as single-strand breaks (SSBs), double-strand breaks (DSBs), base oxidations, and abasic sites, and DNA protein crosslinks are located within an area of 10–20 bp of DNA length [31,32,33]. Compared with C-ion RT, the DNA damage produced by PBT is easier to repair because of an almost complete shift from nonhomologous end joining (NHEJ) to homologous recombination (HR) [34,35]. Jeggo et al. reported that mammalian cells are repaired more often by HR than by NHEJ to process clustered DNA lesions [36].

Fourth, C-ion RT has a strong effect on hypoxic tumors. Low-LET radiation, such as PBT and photons, predominantly induces DNA damage by generating reactive oxygen species (ROS) by indirect radiation effects [37,38]. Hypoxia leads to decreased production of ROS and consequently to reduced DNA damage, resulting in increased radioresistance [37,38]. Although some hypoxia sensitizers are currently in promising clinical trials [39], they remain a major obstacle to radiotherapy. Compared with X-rays, heavy ions have been reported to be less dependent on oxygen concentrations and to reoxygenate tumors earlier in life in vitro and in vivo [40,41]. The clinical results in patients with uterine cancer treated in Japan also indicated that hypoxia radioresistance can be reduced with C-ion RT [42].

## 3. Radiation-Induced Liver Disease (RILD) in Liver Radiotherapy for Hepatocellular Carcinoma (HCC)

According to the current clinical guidelines for HCC from AASLD, EASL, and the Japan Society of Hepatology, the standard locoregional treatment for HCC is resection or radiofrequency ablation. Surgical resection is a well-established treatment, although its application must be carefully selected in elderly patients [43]. In patients who are not candidates for standard locoregional treatment, radiation therapy is an option [8,9,11]. However, radiation therapy has been increasingly used to manage HCC patients who are not eligible for locoregional treatment in real-world clinical practice.

Park et al. reported that the response rates in HCC patients treated with doses <40 Gy, 40–50 Gy, and >50 Gy were 29.2%, 68.6%, and 77.1%, respectively, suggesting that HCC is one of the radiosensitive tumors. They also exhibited it is important to exclude the cases with the presence of extrahepatic metastasis, liver cirrhosis of Child Grade C, tumors occupying more than two-thirds of the entire liver, and a performance status more than three in the commencement of local radiotherapy for HCC [44].

Radiation-induced liver disease (RILD) is the most serious toxicity associated with liver radiotherapy. Although the radiation tolerance of the liver is thought to be <30 Gy in some reports [45], Lawrence et al. reported that all patients who received a mean dose to the whole liver of ≥37 Gy developed radiation hepatitis using 3-D dose–volume analysis [46]. RILD can be classified into classical RILD and nonclassical RILD, and both classical and nonclassical RILD can potentially be life-threatening [47]. Classical RILD is characterized by hepatic veno-occlusive disease (VOD) which is caused structurally by progressive fibrous obliteration of central veins [48] and is defined as elevated alkaline phosphatase (more than two times the upper limit of normal or baseline value) and anicteric hepatomegaly and ascites typically occurring between 2 weeks and 3 months following RT [49]. Classical RILD involves dose-limiting toxicity associated with whole-liver irradiation or conventional RT and is not observed when the mean liver dose is less than 31 Gy [50].

However, SBRT may induce nonclassical RILD, defined as either a worsening of the Child-Pugh Score by two or more points or markedly elevated transaminases (more than five times the upper limit of normal), typically occurring within 3 months of radiation [49]. Patients with chronic hepatic and liver cirrhosis are known to be at risk of developing nonclassical RILD because radiation tolerance is much lower in chronic hepatitis patients than in patients with normal liver function [51]. In large HCC patients, SBRT tends to induce RILD compared with particle beam therapy because of the irradiation dose, which is increased to normal liver tissue [52,53]; thus, SBRT is recommended only for small tumors (generally <5 cm). In contrast, particle beam therapies, such as PBT and C-ion RT, have been applied for large tumors because of their physical advantages, which deposit most of their energy sharply, with a penetration range.

## 4. Clinical Outcomes of Carbon-Ion Radiotherapy (C-Ion RT) for Hepatocellular Carcinoma (HCC)

In 1995, C-ion RT was first used to treat HCC at the National Institute of Radiation Science (NIRS) in Japan. In 2004, the first phase I trial reported the use of NIRS by dose escalation with a total dose of 49.5–79.5 Gy (RBE) in 15 fractions and achieved excellent clinical outcomes (the cumulative local control and OS rates were 81% and 50% at 3 years, respectively, with no severe adverse events) [54]; subsequently, more clinical trials have been conducted. NIRS researchers have conducted several other clinical trials for HCC, and they reported the combined results of phase I and II trials in 2017 [55]. As shown in Table 2, prescription dose was commenced from 54 Gy, 48 Gy, and 48 Gy for the 12-, 8-, and 4-fraction arms, respectively. Median follow-up of 124 Japanese HCC patients was 27.1 (range of 0.9–154.8) months. Clinical trials in Japan included only Japanese patients [55].

The authors defined dose-limiting toxicity as the dose causing acute RILD such as an elevated total bilirubin level >4.0 mg/dL, an aspartate aminotransferase level >800 IU/L, and/or a prothrombin time <25% within 3 months, and the maximum tolerated dose was 52.8 Gy (RBE) in four fractions as the recommended dose regimen for the two phase two studies from 69.6, 58.0, and 52.8 Gy (RBE) in 12, 8, and 4 fractions, respectively [55]. The 3-year local control rates for all lesions and those in the phase 2 trial were 90.3% and 95.5%, respectively (Table 2).

One 3-point increase, acute progression and HCC rupture outside the planning target volume at 3 months after C-ion RT, one 2-point increase in the acute phase, and one 2-point increase in the late phase in the Child-Pugh Score were observed in the phase 2 trial. The percentages of acute- and late-phase Grade 3 reactions in the hematology and skin and late-phase Grade 3 reactions in the pleural effusions were 12% and 13%, 2% and 2%, and 1%, respectively. In these trials, four patients who were Child-Pugh Grade B with other active HCCs died of hepatic failure although it was difficult to conclude whether the cause of death was radiation-induced liver failure, tumor progression, side effects caused by other therapies, or the natural course of their liver disease. The authors suggested that patients with poor liver function, such as those who have Child-Pugh Grade B or worse disease, should be treated with caution when they are undergoing radiotherapy, including C-ion RT (Table 2) [55].

Shibuya et al. reported the results of a phase I trial and a follow-up cohort of Japanese HCC patients with large tumors (3 cm ≤ all < 10 cm) treated with 60 Gy (RBE) in four fractions in 2018 [57]. They used C-ion RT of 60 Gy in four fraction for the treatment of patients with HCC. Median follow-up period of 17 survivors was 24.2 (range of 6.3–43.7) months. The OS rates were 90.5% and 80.0%, and the local control rates were 100% and 92.3%, at 1 and 2 years, respectively. Although two (9.5%) and three (15.7%) patients had worsening Child-Pugh Scores at three and six months, respectively, there was no significant difference in Child-Pugh’s Score at three and six months after C-ion RT compared with those before treatment (*p* = 0.846, Friedman test). These results suggest that C-ion RT with 60 Gy (RBE) in four fractions is safe and can achieve sufficient local control rates in patients with HCC larger than 3 cm, to which SBRT is hardly applicable (Table 2) [57].

Shibuya et al. has reported the results of prospective trials conducted for Japanese HCC patients using 52.8–60 Gy (RBE) in four fractions in 2022 [60]. Median follow-up periods in the survivor group (*n* = 23) and in the whole group (*n* = 35) were 55.1 and 49.0 months, respectively. The cumulative local control rates and OS rates were 76.5% and 76.7%, respectively, at 3 years, with no severe adverse events. The authors revealed that mALBI Grade 2b and Child-Pugh Grade B were the independent poor prognostic factors for OS by the multivariate analysis [60].

Hayashi et al. also suggested that pretreatment Child-Pugh Grade B (*p* = 0.003) and normal liver volume spared from <30 Gy RBE (VS30 < 739 cm^3^) (*p* = 0.009) are significant risk factors for RILD following C-ion RT for Japanese patients with HCC [65]. They analyzed HCC patients who received a radiation dose of 60 Gy in four fractions. Median follow-up period was 9.7 (range of 2.3–41.1) months.

Liver function in Child-Pugh Grade B patients ranges from tolerable to difficult to treat with local therapy. Hiroshima et al. reported the safety and effectiveness of C-ion RT in Japanese HCC patients with Child-Pugh Grade B [62]. Total dose and dose fraction were 45 or 48 Gy/2 fractions, in other words 22.5 or 24 Gy/fraction, respectively, when no organ at risk, such was in proximity, and 52.8 or 60 Gy/4 fractions, in other words 13.2 or 15 Gy/fraction, when it was at risk. Median follow-up period was 20.5 (range of 2.7–108) months [62]. The OS was 80.4% and 46.0%, and the local control rates were 96.4% and 96.4% at 1 and 2 years, respectively. There were no acute or late adverse events of grade ≥4, and three and two patients had Child-Pugh Scores that increased by more than two points in the acute and late phases, respectively. C-ion RT has no relationship with the mean irradiated liver dose (MLD) or exacerbation of Child-Pugh Score in the late phase (*p* = 0.206), although the Child-Pugh Score worsened by one when the MLD exceeded 11 Gy in SBRT [68]. Compared with SBRT, C-ion RT can be administered at sufficient doses to increase HCC radiosensitivity without increasing the MLD because of its improved dose distribution.

Shiba et al. reported the results of retrospective trials conducted in 2017 and 2018 [43,56], and in these trials they enrolled 31 HCC patients who were all over 80 years old and 22 patients with sarcopenia or 46 patients without sarcopenia. Japanese patients received C-ion RT with 52.8 Gy (RBE) or 60.0 Gy (RBE) in four fractions for usual cases and 60.0 Gy in 12 fractions for close-to-gastrointestinal tract cases [43]. The dose fractionation schedule was 52.8 Gy/4 fractions in 37 Japanese patients and 60 Gy/4 fractions in 31 Japanese patients [56]. Patients received C-ion RT once daily for four days per week (from Tuesday to Friday) [56]. The median follow-up period of all patients was 23.2 (range of 8.4–55.3) months [43], or the median follow-up period of patients was 33.5 months [56].

The cumulative OS rate for patients with HCC over 80 years were 82.3% at 2 years, and for patients with HCC with sarcopenia and without sarcopenia, it was 66% and 77% at 3 years, respectively (Table 2) [43,56]. Late Grade 3 encephalopathy occurred in 10% of patients over 80 years of age who had HCC with no Grade 4 or greater toxicity, and late Grade 3 encephalopathy occurred in 9% and 4% of patients who had sarcopenia and nonsarcopenia, respectively, with no Grade 4 or greater toxicity. First, the authors suggested that C-ion RT may become an alternative treatment option for elderly HCC patients for whom resection or radiofrequency ablation are not a viable choice because of their good treatment outcome and short-term treatment period [43]. Second, the authors revealed that sarcopenia was not a prognostic factor for HCC patients treated with C-ion RT but rather a prognostic factor for patients with HCC treated with hepatic resection [69,70] because the lower severe toxicity and no fasting duration during C-ion RT may have been associated with the same efficacy in sarcopenia patients as in nonsarcopenia patients [56].

Shiba et al. reported the results of retrospective trials including 11 Japanese patients who had locally advanced HCC, with cumulative local control rates of 78% and OS rates of 64% (median OS: 36.4 months) at 3 years. This clinical outcome is better than that of other anticancer treatments (median OS: 8.1–9.9 month with sorafenib; 3-year OS: 42% with TACE; and 3-year OS: 13–68% with surgery) [71,72,73,74]. Late grade 3 bone fracture occurred in 9% of those patients, with no grade 4 or higher toxicity [59]. Shiba et al. suggested that high-dose C-ion RT administration may result in long-term, local recurrence-free survival [59]. The C-ion RT dose of 52.8 Gy/4 fractions or 60.0 Gy/4 fractions was delivered for standard Japanese patients, and the dose of 60.0 Gy/12 fractions was delivered for close-to-gastrointestinal-tract patients. Median follow-up period after C-ion RT was 36.4 months. The authors concluded that C-ion RT was potentially preferable to liver resection in locally advanced HCC patients because of its comparable or favorable OS [59].

Kaneko et al. also reported the results of retrospective trials including 76 Japanese patients who had locally advanced HCC and exhibited better outcomes with no grade 4 or higher toxicity [64]. Their retrospective cohort study evaluated HCC patients with macroscopic vascular invasion treated using C-ion RT with a dose of 45.0–48.0 Gy/2 fractions or 52.8–60.0 Gy/4 fractions. The median follow-up period was 27.9 (range of 1.5–180.4) months [64].

Yasuda et al. reported the safety and effectiveness of C-ion RT with two fractions in Japanese HCC patients at a total dose of 45 Gy (RBE) [58], although, in long-term observations in the last phase 1/2 clinical trial of two-fraction C-ion RT using a dose of 38.8 Gy (RBE) or less, the local control rates were not as good as those of previous clinical trials, which was attributed to an inadequate dose. The median follow-up period was 54 (range, 7–103) months [58]. The local control rates and OS rates at 5 years were 91% and 45%, respectively. These data are comparable with those of previous clinical trials in which 15, 12, 8, and 4 fractions were used [54,55]. Moreover, these data are comparable with those of a phase 2 study of SBRT with three to six fractions in patients with solitary HCC 4 cm or less in diameter [75]. In the present study, no other grade 3 or higher toxicity was observed in the late phase. Thus, two-fraction C-ion RT is a minimally invasive therapeutic option (Table 2).

In Germany, Hoegen-Saßmannshausen et al. first reported the safety and effectiveness of a single-arm, single-center, phase I dose-finding study using the biological RBE-model local effect model I (LEM I) and active raster scanning as delivery methods in Europe [66]. C-ion RT was performed every other day in four fractions (8.1–10.5 Gy) (total doses of 32.4–42.0 Gy), according to a Japanese model [54,55,57,60]. The median follow-up period was 23 months [66]. In this study, the heterogeneity of population was not described [66]. The authors reported no dose-limiting toxicity (DLT), and practically no acute toxicity greater than CTCAE grade II occurred. Although OS (75% at 1 year and 22% at 3 years) was significantly shorter than published data (90.3–97% at 1 year and 50.2–76.7% at 3 years) for more frail patients with lower performance status, local control rates were excellent, without a single local recurrence (Table 2) [55,58,60,64].

In China, Hong et al. first reported C-ion RT with the pencil beam scanning technique [61]. Fifty-eight Chinese HCC patients received doses of 50–70 Gy in 10 fractions, and 32 received 60–67.5 Gy in 15 fractions, according to the tumor location and normal tissue constraints. The median follow-up period was 28.6 (range of 5.7–74.6) months [61]. The OS rates at 1, 3, and 5 years were 91.3%, 81.9%, and 67.1%, the local control rates were 100%, 94.4%, and 94.4%, and the PSFs were 73.6%, 59.2%, and 37.0%, respectively. Two patients (8.7%) experienced grade 3 leukocytopenia as acute toxicity, and two patients (8.7%) experienced grade 3 stomach bleeding as a late toxicity. The authors concluded that C-ion RT with the pencil beam scanning technique up to 70 Gy in 10 fractions over a 2–week period was safe and effective for treating HCC in a phase I trial in 2022 [61].

Zhang et al. also retrospectively reported the results of C-ion RT with the pencil beam scanning technique using RBE-weighted doses (DRBE) of 50–70 Gy in 10 fractions or 60–67.5 Gy in 15 fractions for 90 Chinese HCC patients [63]. The median follow-up period was 28.6 (range of 5.7–74.6) months. The OS rates at 1, 3, and 5 years were 97.8%, 83.3%, and 75.4%, respectively. However, two patients died after C-ion RT because of late toxicity caused by stomach bleeding attributed to cirrhotic portal hypertension and repeated jaundice attributed to bile duct structure. There were two patients (2.2%) with grade 3 or higher biliary stricture/obstruction requiring intervention in this study, which was consistent with the findings of previous studies following PRT [76]. The authors avoid high doses to the central biliary tree and adopt more moderate regimens of 15 fractions for tumors near the main bile ducts [63].

## 5. Comparison of Carbon-Ion Radiotherapy (C-Ion RT)and Other Therapies for Hepatocellular Carcinoma (HCC)

Stereotactic radiosurgery (SRS), which couples X-rays with a stereotactic guiding device, was first performed to treat brain tumors by Leksell in 1951 [77]. Although SRS has already proven to be a safe and effective way to treat acoustic neuromas and the solid component of craniopharyngiomas [78], radiation therapy by medical X-ray applications for the treatment of HCC was limited to palliation because there was some concern of the risk of liver damage due to suboptimal image guidance and inadequate motion management until the 1980s.

Recently, SBRT, which makes use of the principles of SRS with intensity-modulated radiotherapy (IMRT), which is an advanced conformal radiotherapy method that yields highly conformal dose distributions than 3D conformal techniques, resulting in improved toxicity outcomes and local control [79] and motion management strategies [17], has provided excellent symptom palliation and local control with toxicity reduction. Motion management strategies commonly use phase-based gating [80,81] and displacement-based gating [80,81] for targets that move with respiration. The phase-based gating is based on a quantity derived from its prior shape, and displacement-based gating is based directly on the current value of the respiration trace [17,81]. SBRT is an effective treatment concept in which optimizes the gating duty cycle to high doses of radiation delivery efficiency in some radiation fractions, resulting in complication reduction.

Several studies from Gunma University have evaluated C-ion RT in comparison with other treatment modalities for HCC.

Abe et al. compared dose–volume parameters for C-ion RT versus SBRT in terms of target volume coverage and reducing the number of organs at risk [82]. The minimum doses covering 90% of the planning target volume (PTV D90) for C-ion RT and SBRT were 59.6 ± 0.2 Gy (RBE) and 56.6 ± 0.3 Gy (*p* < 0.05), respectively, and the conformity indices (CI) were 0.79 ± 0.06 and 0.37 ± 0.02 (*p* < 0.05), respectively. These data indicate that C-ion RT is superior to SBRT in generating a more conformal dose distribution. The mean liver dose (MLD) for normal tissue sparing for C-ion RT and SBRT patients was 8.1 ± 1.4 Gy (RBE) and 16.1 ± 2.5 Gy (*p* < 0.05), respectively. The normal liver volumes receiving more than 5 Gy (RBE) (V5) and 20 Gy (RBE) (V20) were greater in the SBRT group than in the C-ion RT group. These data indicate that C-ion RT may have advantages for patients with poor liver function because of its capacity to decrease low-dose scattering to the normal liver compared with SBRT [82].

Similarly, Shiba et al. suggested that C-ion RT has a superior dose distribution than IMRT when treating locally advanced HCC involving a major branch of the portal or hepatic vein, including PVTT or IVCTT, which are difficult to treat with IMRT at 60 Gy owing to the high risk of developing RILD and produce a lower dose to the liver, indicating that C-ion RT may expand the indications for the treatment of locally advanced HCC [83].

Compared to SBRT, there are the limited institutions where C-ion RT and PBT are available in several prefectures of Japan. These are also limitations of these methods. The safety and efficacy of C-ion RT for HCC has mainly been studied in Japan, China, Italy, and Germany [66,84,85,86]. The representative data were shown in Table 2. PBT for HCC has been developed in Hong Kong, Japan, United States, etc. [87,88,89,90,91]. We should perform more collaboration with other liver centers outside Japan in Europe and United States to assess for improvement in prognosis with C-ion RT.

Fujita et al. compared C-ion RT with RFA as an initial treatment for early stage HCC [92]. Their retrospective analysis showed that C-ion RT resulted in a lower cumulative intrasubsegmental recurrence rate, while yielding a comparable cumulative local recurrence rate, PFS, and OS compared with RFA in patients with a single lesion ≤5 cm or two to three lesions ≤3 cm HCC. These findings suggested that C-ion RT may represent an effective treatment option for early stage HCC when RFA is not indicated.

Patients who decline surgery and RFA have usually been treated with TACE according to various guidelines [8,9,10,11]. Thus, the authors compared C-ion RT and TACE [82,83]. Shiba et al. reported more favorable clinical outcomes for C-ion RT than for TACE in patients. The 3-year OS, local control rates, and PFS rates in C-ion RT versus TACE were 88% versus 58% (*p* < 0.05), 80% versus 26% (*p* < 0.01), and 51% versus 15% (*p* < 0.05), respectively, with single HCC as a primary treatment after patient characteristics were matched by propensity score matching. The three-year OS rates of TACE patients range from 50% to 58%, which is similar to the OS rate range reported in this study [93,94]. Moreover, the number of patients who progressed to a worse Child-Pugh Grade was significantly greater in the TACE group than that of in the C-ion RT group (*p* < 0.01) [95]. These data suggest that liver function preservation with C-ion RT may also contribute to favorable OS.

In Japan, it costs 3,000,000 JPY, 3,000,000 JPY, and 500,000 JPY for C-ion RT for unresectable HCC (>40 mm), PBT for unresectable HCC (>40 mm), and SBRT for HCC (<50 mm and tumor number <3), respectively. However, Okazaki et al. also reported that C-ion RT is a cost-effective treatment option compared with TACE for patients with localized HCC unsuitable for surgical resection on the basis that a second C-ion RT treatment on the same organ within 2 years was performed for free, because the superior local control and minimal adverse effects of C-ion RT may reduce posttreatment expenses [96]. C-ion RT seems to be indicated for patients who are likely to decline TACE [67]. Thus, TACE is considered the most likely replacement by the C-ion RT [97,98].

Although radiotherapy has long been known for its immunogenicity, the benefits of combining it with immune checkpoint inhibitors (ICIs) have yet to be proven clinically [8,9]. The HCC microenvironment, including tertiary lymphoid structures, plays a role in the efficacy of ICI therapies [99,100]. The combination of radiation and ICIs may take advantage of their complementary mechanisms [101,102]. The combination of ICIs and C-ion RT may be a treatment option for patients with localized and advanced HCC, as may combination therapy with multi-kinase inhibitors and C-ion RT [103,104]. However, further studies are needed.

## 6. Conclusions

HCC is a radiosensitive cancer, and C-ion RT, a type of particle beam therapy, has shown excellent local control for HCC recurrence. C-ion RT may be better for certain patients with HCC than other treatment, such as TACE. International collaboration of the study should be performed to resolve the potential problems in the C-ion RT for HCC.

## Figures and Tables

**Table 1:** Representative external beam therapies for hepatocellular carcinoma.

Photon Beam Therapies	Particle Beam Therapies
*X-rays* *and gamma rays*	*Beams of protons and heavy ions*
General high-energy radiation	Carbon-ion radiotherapy (C-ion RT)
Three-dimensional conformal radiation therapy	Proton beam therapy (PBT)
Intensity-modulated radiation therapy (IMRT)	
Volumetric-modulated arc therapy (VMAT)	
Image-guided radiotherapy (IGRT)	
Stereotactic body radiation therapy (SBRT)	

**Table 2:** Efficacy and adverse events by carbon-ion radiotherapy (C-ion RT) for hepatocellular carcinoma.

City, Country	Year	Study	Participants	Results, Efficacy	Adverse Events
Chiba, Japan [55]	2017	Retrospective study [Phase I and II studies]	*N* = 124 (68 (37–84) years, 71% male/69 (46–84) years, 68% male); Single HCC (77%/74%); Child-Pugh Grade A, B (77%, 23%/80%, 20%); BCLC stage A, B, C (27%, 13%, 60%/20%, 14%, 66%); Main tumor size (mm) 40 (10–120)/37 (12–86); PS 0–1, 2 (87%, 13%/89%, 11%); Treatment-naïve (52%/64%)	Prescription doses, 69.6, 58.0, and 52.8 Gy (RBE) in 12, 8, and 4 fractions/52.8 Gy (RBE) in 4 fractions; Follow-up duration, 27.1 (0.9–154.8) months; OS rates, 1, 3 and 5 years: 90.3%, 50.0% and 25.0%; LC rates, 1, 3 and 5 years: 94.7%, 91.4% and 90.0%	Major complications, 0%; White blood cells, G3, 1%; Hemoglobin, G3, 1%; Platelets, G3, 10%; Skin, G3, 2%; Skin, G2, 7%; Gastrointestinal tract, G2, 2%; Child-Pugh Score, +2, 3%; Child-Pugh Score, +3, 1%
Maebashi, Japan [43]	2017	Retrospective study	*N* = 31 (83 (80–95) years, 71% male); No intrahepatic metastasis or distant metastasis; Child-Pugh Grade A, B (87%,13%); BCLC stage A, B, C (42%, 3%, 55%); Main tumor size (mm) 45 (15–93); PS 0, 1, 2 (55%, 39%, 6%); Prior therapy: TACE (10), RFA with TACE (2), TAI (1)	Prescription doses, 52.8 Gy (RBE) in 4 fractions or 60.0 Gy (RBE) in 4 fractions, 60.0 Gy (RBE) in 12 fractions for close-to-gastrointestinal tract cases, which were defined as a distance of < 1 cm between tumor and gastrointestinal tract; Follow-up duration, 23.2 (8.4–55.3) months; OS rates, 2 years: 82.3%; LC rates, 2 years: 89.2%	Major complications, 0%; Dermatitis, G1, 94%; Pneumonitis, G1, 26%, Encephalopathy, G1, 3%; Child-Pugh Score, +2, 3%
Maebashi, Japan [56]	2018	Retrospective study	*N* = 68 (sarcopenia/nonsarcopenia, 22/46, 77 (57–95) years/74 (45–90) years, 50% male/65% male); Single HCC and no direct infiltration of the gastrointestinal tract, any intrahepatic metastasis, or distant metastasis; Main tumor size (mm) 30 (12–90)/36 (9–77); PS 0, 1, 2 (55%, 45%, 0%)/(72%/24%/4%)	Prescription doses, 52.8 Gy (RBE) in 4 fraction or 60.0 Gy (RBE) in 4 fractions; Follow-up duration, 33.5 (3.9–83.1) months; OS rates, 3 years: 66% in sarcopenia/77% in nonsarcopenia; LC rates, 3 years: 81% in sarcopenia/72% in nonsarcopenia	Major complications, 0%; ALT G2, 5%; ALT G1 18%; Dermatitis G1, 86% (in sarcopenia group)/ALT G2, 2%; ALT G1 2%; Dermatitis G1, 87% (in nonsarcopenia group)
Maebashi, Japan [57]	2019	Phase I trial and the follow-up cohort	*N* = 21 (76 (58–88) years, 67% male); Single HCC (95%), 3 cm ≤ All HCC < 10 cm; Child-Pugh Grade A, 100%; UICC stage I/II/IIIa/IIIb, 71%/14%/5%/10%; Main tumor size (mm) 48 (30–78); PS 0, 1, 2 (62%, 29%, 9%); Treatment-naïve, 67%	Prescription doses, 60 Gy in 4 fractions; Follow-up duration, 24.2 (6.3–43.7) months; OS rates, 1 and 2 years: 90.5% and 80.0%; LC rates, 1 and 2 years: 100% and 92.3%	Major complications, 0%; Gastrointestinal G2, 5%; Investigation G2, 14%
Chiba, Japan [58]	2019	Retrospective study	*N* = 57 (75 (49–89) years, 58% male); Single HCC (98%); Child-Pugh Grade A, B (89%, 11%); Main tumor size (mm) 33 (13–95); PS 0, 1, 2 (68%, 30%, 2%); Treatment-naïve, 72%	Prescription doses, 45 Gy in 2 fraction; Follow-up duration, 54 (7–103) months; OS rates, 1, 3 and 5 years: 97%, 67% and 45%; LC rates, 1, 3 and 5 years: 98%, 91% and 91%	Major complications, 0%; Skin G3, 4%; Skin G2, 7%; Liver G2, 9%; Skin G1, 86%; Liver G1, 28%; Lung G1, 21%
Maebashi, Japan [59]	2020	Retrospective study	*N* = 11 (76 (57–86) years, 82% male); Child-Pugh Grade A, B (91%, 9%); BCLC stage A, B, C (18%, 0%, 82%); Main tumor size (mm) 53 (27–119); PS = <2 (100%)	Prescription doses, 52.8 Gy in 4 fractions or 60.0 Gy in 4 fractions, 60.0 Gy/in 12 fractions for close-to-gastrointestinal tract cases; Follow-up duration, 36.4 (4.3–86.2) months; OS rates, 3 years: 64%; LC rates, 3 years: 78%	Major complications, 0%; Dermatitis G1, 82%; Pneumonitis G1, 28%; Acsitis G1, 18%
Maebashi, Japan [60]	2021	Prospective study	*N* = 35 (75 (57–85) years, 51% male); All HCC ≤ 10 cm; Child-Pugh Grade A, B 83%, 17%; ALBI 1/2a/2b 26%/31%/43%; Main tumor size (mm) 35 (12–77); PS 0, 1, 2 (68%, 29%, 3%); Treatment-naïve, 57%	Prescription doses, 52.8 Gy in 4 fractions (49%) or 60 Gy in 4 fractions (51%); Follow-up duration, 49 (4–62.4) months; OS rates, 2 years: 82.8%; LC rates, 2 years: 92.6%	Major complications, 0%; Ascites G2, 3%; AST G2, 3%; Hypoalbuminemia G2, 3%
Shanghai, China [61]	2023	Phase I study	*N* = 23 (57 (28–76) years, 87% male); All HCC ≤ 12 cm; Child-Pugh Grade A 100%, 17%; BCLC stage 0, A, B, C (4%, 0%, 57%, 43%); Main tumor size (mm) 43 (17–85); PS 0, 1, 2 (52%, 48%, 0%)	Prescription doses, 55 Gy in 10 fractions, 60 Gy in 10 fractions, 65 in 10 fractions, or 70 Gy in 10 fractions, 22%/26%/35%/17%; Follow-up duration, 56.1 (5.7–74.4) months; OS rates, 1, 3 and 5 years: 91.3%, 81.9%, and 67.1%; LC rates, 1, 3 and 5 years: 100%, 94.4%, and 73.6%	Major complications, 0%; Leukocytopenia G3, 9%; Leukocytopenia G2, 9%; Neutrocytopenia G2, 26%; Thrombocytopenia G2, 9%; Bilirubin increase G2, 4.3%; Albumin decrease G2, 4.3%; Skin injury G1, 44%; Abdominal pain G1,17%; Leukocytopenia G1, 17%; Neutrocytopenia G1, 13%; Thrombocytopenia G1, 9%; ALP G1, 9% increase; Albumin decrease G1, 9%
Chiba, Japan [62]	2023	Retrospective study	*N* = 58 (57 (28–76) years, 87% male); 1~4, HCC ≤ 3 irradiation fields; Child-Pugh Grade B, 100%, score 7/8/9, 72%/22%/6%; ALBI 1/2a/2b/3 2%/12%/79%/7%; Main tumor size (mm) 32 (7–135); PS 0, 1, 2 (74%, 21%, 5%); Treatment-naïve, 24%	Prescription doses, 45 or 48 Gy in 2 fractions, 52.8 or 60 Gy in 4 fractions (when some organ at risk, such as the gastrointestinal (GI) tracts); Follow-up duration, 20.5 (2.7–108) months; OS rates, 1 and 2 years: 80.4% and 46.0%; LC rates, 1 and 2 years: 96.4% and 96.4%	Major complications, 0%. Child-Pugh Score increased more than 2 points in the acute and late phase were 3 and 2 patients, respectively.
Shanghai, China [63]	2023	Retrospective study	*N* = 90 (58.5 (28–87) years, 84% male); Single HCC (74%); Child-Pugh Grade A/B, 99%/1%; BCLC stage 0, A, B, C (2%, 7%, 46%, 46%); Main tumor size (mm) 46 (1.6–15.5); PS 0, 1, 2 (52%, 48%, 0%); PS 0, 1, 2 (68%, 32%, 0%)	Prescription doses, 50–70 Gy in 10 fractions (64%), 60–67.5 Gy in 15 fractions (36%); Follow-up duration, 28.6 (5.7–74.6) months; OS rates, 1, 2 and 3 years: 97.8%, 83.3% and 75.4%; LC rates, 1, 2 and 3 years: 96.4%, 96.4% and 93.1%	Death, 2; Leucopenia G3–4, 6.7%; Thrombocytopenia G3–4, 3.3%; Leucopenia G1–2, 38.9%; Thrombocytopenia G1–2, 25.6%; Hyperbilirubinemia G1–2, 2.2%; Hypoalbuminemia G1–2, 10%; γ-glutamyltransferase increase G1–2, 2.2%; Alkaline phosphatase increases G1–2, 6.7%; Dysphagia G1–2, 2.2%; Dermatitis radiation G1–2, 35.6%; Abdominal pain G1–2, 20%
Chiba, Japan [64]	2024	Retrospective cohort study	*N* = 76 (71 (45–86) years, 76% male); 1~4 HCC; Child-Pugh Grade A/B, 89%/11%; ALBI 1/2 55%/45%; Main tumor size (mm) 46 (15–130); PS 0, 1, 2 (72%, 23%, 4%); Treatment-naïve, 52.6%	Prescription doses, 45.0~48.0 Gy in 2 fractions, 52.8~60.0 Gy in 4 fractions; Follow-up duration, 27.9 (1.5–180.4) months; OS rates, 2 and 3 years: 70.0% and 50.2%; Local recurrence rates, 2 and 3 years: 8.9% and 10.7%	Major complications, 0%; Dermatitis G3, 3%; Hepatobiliary disorder G3, 3%; Dermatitis G1, 1%; Hepatobiliary disorder G1, 4%
Osaka, Japan [65]	2024	Retrospective study	*N* = 108 (76 (47–95) years, 71% male); 1~5 HCC; Child-Pugh Grade A/B, 91%/9%; Main tumor size (mm) 41 (5–160); PS 0, 1, 2 (79%, 17%, 5%); Treatment-naïve, 66.6%	Prescription doses, 60 Gy in 4 fractions; Follow-up duration, 9.7 (2.3–41.1) months; OS rates, N/A; LC rates, N/A	Major complications, 0%. The pretreatment Child-Pugh Grade B (HR = 6.90; *p* = 0.003) and normal liver volume spared from <30 Gy RBE (HR = 5.22; *p* = 0.009) were significant risk factors for RILD.
Heidelberg, Germany [66]	2024	Phase I study	*N* = 20 (74.7 (55.7–83.6) years, 75% male); 1~5 HCC; Child-Pugh Grade A/B, 70%/10%; BCLC stage 0, A, B, C (15%, 30%, 55%, 0%); PS 0, 1, 2 (45%, 50%, 5%; Treatment-naïve, 66.6%	Prescription doses, 32.0~42.0 Gy in 4 fractions; Follow-up duration, 23.0 months; OS rates, 1, 2, 3, and 4 years: 75%, 64%, 22%, and 15%; LC rates, 1, 2, and 3 years: 59%, 43%, and 43%	Major complications, 0%; No toxicity
Taipei, Taiwan [67]	2025	Phase I study	*N* = 1 (72-year-old man); 25 × 23 mm HCC in the right hepatic lobe; Clinical staging, T1bN0M0	Prescription doses, 52.8 Gy (RBE) in 4 fractions; Follow-up duration, 3 months; Complete response	Major complications, 0%; No adverse events

HCC, hepatocellular carcinoma; RBE, relative biological effectiveness; OS, overall survival; LC, local control; TACE, transarterial chemoembolization; RFA, radiofrequency ablation; TAI, transarterial infusion; UICC stage, Union for International Cancer Control TNM (“Tumor”, “Nodes”, and “Metastases”) cancer staging system; RILD, radiation-induced liver damage; HR, hazard ratio; N/A, not available.

## Data Availability

Not applicable.
